# Subtypes Distribution and Frequency of *Blastocystis* sp. Isolated from Diarrheic and Non-diarrheic Patients

**Published:** 2017

**Authors:** Nahid JALALLOU, Shahrokh IRAVANI, Mostafa REZAEIAN, Atefe ALINAGHIZADE, Hamed MIRJALALI

**Affiliations:** 1. Dept. of Laboratory Sciences, School of Paramedicine, AJA University, Tehran, Iran; 2. AJA Cancer Research Center (ACRC), AJA University of Medical Sciences, Tehran, Iran; 3. Dept. of Medical Parasitology and Mycology, School of Public Health, Tehran University of Medical Sciences, Tehran, Iran; 4. Foodborne and Waterborne Diseases Research Center, Research Institute for Gastroenterology and Liver Diseases, Shahid Beheshti University of Medical Sciences, Tehran, Iran

**Keywords:** *Blastocystis* sp., 18S rDNA, Subtyping, Iran

## Abstract

**Background:**

*Blastocystis* is one of the most common parasites, reported from both human and animals. This parasite is more prevalent in regions with low levels of hygiene, close contact with animal and unsuitable disposal systems. The aim of the study was to subtype *Blastocystis* sp., isolated from diarrheic and non-diarrheic patients using sequencing of 18S ribosomal DNA.

**Methods:**

Totally, 300 stool samples were collected from diarrheic and nondiarrheic patients referred to Imam Reza Hospital, Tehran from Apr to Aug 2015. All samples were concentrated using conventional Formalin – ether technique and recognized under light microscope. The fresh stool samples were also cultivated in clotted fetal bovine medium and examined for growing of *Blastocystis* every 48 h with direct smear slides for 10 d. DNA extraction was performed on all positive samples. Amplified DNA fragment of 18S rDNA was sequenced and compared with reference genes, previously deposited in Genbank database.

**Results:**

The number of diarrheic and non-diarrheic patients participated in the study was 134 (44.66%) and 166 (55.34%), respectively. Three subtypes 1, 2, 3 were identified from positive samples. Subtype 2 was the most prevalent (36.5%) followed by subtype 1 (33.3%) and subtype 3 (30.2%). There were no mixed subtypes. Furthermore, the most prevalent subtypes in diarrheic and non-diarrheic patients were subtype 2 (39.28%) and subtype 1 (37.14%), respectively.

**Conclusion:**

*Blastocystis* sp., is one of the most prevalent unicellular parasites among diarrheic and non-diarrheic patients. Indeed, ST2 was the most prevalent subtype particularly in those samples collected from diarrheic patients.

## Introduction

*Blastocystis* sp. is a controversially unicellular parasite reporting from gastrointestinal tract of symptomatic and asymptomatic subjects (1, 2). This unicellular protozoan is one of the most common parasites, all over the world and is reported from both human and animals (3). Fecal-oral is known as the main rout of transmission of the parasite, but, the actual source of transmission has not been clearly known (4–6). The prevalence rate of *Blastocystis* is different according to the study area and population as well as the method of detection (7). However, the parasite is more prevalent in regions with low levels of hygiene, close contact with animal and unsuitable disposal systems (3).

Although, the pathogenic role of *Blastocystis* is vague, an eventual linkage has been suggested between symptoms and different subtypes of *Blastocystis* (8). Several studies have described correlation between existence of the parasite and some disorders such as abdominal pain (9, 10), diarrhea and constipation (11, 12), Inflammatory Bowel Syndrome (IBS) (13–15) and recently urticarial (16, 17).

Based on polymorphic region of Small Subunit ribosomal DNA (SSU rDNA), 17 subtypes have been introduced in which nine of them have been reported from human (18, 19). Therefore, this discriminative region of rDNA has used for analysis of different epidemiological studies (20, 21).

In Iran, description of molecular distribution of *Blastocystis* subtypes was carried out around different regions of the country. All studies in Iran were performed using PCR-RFLP (22) and sequences tagged-size primers (STS) (23, 24). Primers selection, number and place of mutations, and mixed subtypes are the limitations of RFLP approach (3). On the other hand, STS primers are less sensitivity compared to amplifying 18S rDNA of *Blastocystis* (7).

The present study is the first one that frequency and subtype distribution of *Blastocystis* sp., was evaluated using amplifying of SSU rDNA in diarrheic and non-diarrheic patients in Iran.

## Materials and Methods

### Sampling

Three hundred stool samples were collected from diarrheic and non-diarrheic patients referred to Imam Reza Hospital, Tehran, from Apr to Aug 2015. All samples were freshly transferred to the laboratory and were divided into two portions; a portion for microscopical identification and the other portion for cultivation. All samples were concentrated using conventional formalin – ether technique and recognized under light microscope.

The study was approved by Ethics Committee of the university and informed consent was taken from all patients.

### Culture

The fresh stool samples were cultivated in LE medium as were described (25) with some modification. Briefly, autoclaved 1ml Locke’s solution was added to 2ml slant clotted fetal bovine serum in a screw-cap tube. Then, 200mg from each stool samples together starch and 4mg/ml streptomycin were added to each cultivation tube. All samples were incubated at 37 °C and anaerobic condition for 10 days. Each culture was examined for growing of *Blastocystis* every 48 h with direct smear slides for 10 days. The cultures that the parasite was not seen in them after 10 days, reported as negative.

### DNA extraction PCR and subtyping

Totally, 250 μl of liquid phase of medium of each positive sample was transferred to 1.5 ml tube and was centrifuged at 5000 rpm for 5 min. Then, supernatant was discarded, and the pellet was introduced to i-genomic stool DNA Extraction kit (TECH DRAGON LIMITED, Hong Kong). Purified DNA was stored at −20 °C until use.

DNA amplification was performed on all positive samples using 18S rDNA specific primers, described elsewhere including F1 (5′-GGA GGT AGT GAC AAT AAA TC-3′) (26) and BHCRseq3 (5′-TAA GAC TAC GAG GGT ATC TA-3′) (7). The primers amplified a 550 bp fragment of SSU rDNA of *Blastocystis* sp., specifically according to following protocol: denaturation at 95 °C for 7 min, 35 cycles at 94 °C for 60 sec, 56 °C for 45 sec, 72 °C for 45 sec, followed by a final extension at 72 °C for 7 min.

Subsequently, 10 μL of each PCR product was electrophoresed on a 1.5% agarose gel in TBE (0.09M Tris, 0.09 M boric acid, 2 mM EDTA) stained with 0.5 μg/mL ethidium bromide and visualized using a UV Transilluminator.

Finally, an ABI (3130) sequencer (California, USA) sequenced 20 μL of all PCR products and the results were compared with those available in GenBank database.

## Results

Seventy one (23.7%) and 229 (76.3%) of studied individuals were female and male, respectively. Of 300 stool samples, *Blastocystis* sp. was observed from 55 (18.3%) and 63 (21%) samples of formalin-ether concentration and cultures, respectively. The mean± Std. Deviation of age of enrolled patients was 42.77± 17.53 and subjects with age ≥ 70 yr old were seen infected with *Blastocystis* sp., more than other patients.

The number of diarrheic and non-diarrheic patients included in the study was 134 (44.66%) and 166 (55.34%), respectively (Table 1).

**Table 1:** Frequency of *Blastocystis* spp., identified by conventional formalin-ether and Cultivation in diarrheic and non-diarrheic patients

**Variable**	**Formalin – ether no. (%)**		**Cultivation no. (%)**	
	**Positive**	**Negative**	**Positive**	**Negative**
Diarrheic patients	26 (19.41%)	108 (80.59%)	28 (20.9%)	106 (79.1%)
Non-diarrheic patients	29 (17.47%)	137 (82.53%)	35 (21.09%)	131 (78.91%)
Total	55(18.3)	245(81.7)	63(21)	237(79)

All sequences were edited and then compared with sequences that were already available in GenBank database. Three subtypes 1, 2, 3 were identified from all positive samples using PCR and sequencing of the 18S rDNA. Subtype 2 was the most prevalent (36.5%) followed by subtype 1 (33.3%) and subtype 3 (30.2%). There were no mixed subtypes and the most prevalent subtypes in diarrheic and non-diarrheic patients were subtype 2 (39.28%) and subtype 1 (37.14%), respectively (Table 2).

**Table 2:** Subtypes frequency of *Blastocystis* spp., isolated from diarrheic and non-diarrheic patients

**Variable**	**Diarrheic patients**	**Non diarrheic patients**	**Total No. (%)**
Subtype 1	8	13	21 (33.3)
Subtype 2	11	12	23 (36.5)
Subtype 3	9	10	19 (30.2)
Mixed subtypes	none	none	None
Total	28	35	63

## Discussion

*Blastocystis* spp. is one of the most prevalent parasites, frequently reported from human and animals (3). Several studies have shown moderate to high prevalence rate of this parasite in Iran (27, 28), but recently, have been seen a growing interest into study on genetic diversity and heterogeneity of *Blastocystis* in symptomatic and asymptomatic patients.

In our study, *Blastocystis* was observed in 21% of the samples, cultivated in LE medium. Epidemiological studies in Iran have shown that the prevalence rate of the parasite is from about 2% to 29% in both symptomatic and/or asymptomatic individuals. Almost all of these studies have been performed using direct smear slide and iodine staining, while cultivation of *Blastocystis* increases the sensitivity of detection. Our finding is in agreement with Moosavi and colleagues study that showed higher prevalence of Blastocystis in culture medium compared to microscopic method (23). In another study, Badparva and colleagues reported prevalence rate 6.5% among patients referred to hospital (24) that is more likely related to the subject that cultivation increases the chance of detection of *Blastocystis*.

In this study, subtypes 1, 2 and 3 were obtained from diarrheic and none-diarrheic patients. These subtypes are the most prevalent subtypes reported from other studies in Iran as well as other countries (21, 29). A study on Indian population showed that ST3 was the most common subtypes followed by ST1 (20). In Libya, ST1 was the predominant subtype, while ST2 and ST3 had lesser prevalence (30). In our study, ST2 was the predominant followed by ST1 and ST3. Based on PCR-RFLP approaches, ST1 was the most common subtype in south of Iran (22) while, in another study based on STS primers, ST3 was the dominant subtype (23, 24).

Some studies have showed that the prevalence of ST1 in symptomatic and IBS patients is higher than other STs and thus, this subtype was described that as a pathogenic subtype (31, 32), but a clear association between *Blastocystis* STs and different clinical manifestations has not still been established. However, in our study ST2 was the dominant subtype among patients with diarrhea, while ST1 was the most common subtype in none-diarrheic patients. Although, ST1 was not reported as dominant subtype in Lebanese patients, but ST1 was significantly more prevalent among GI patients (33). On the other hand, potential linkage between ST2 and asymptomatic patients was previously described in Turkey (34).

In the current study, despite of majority of ST2 in diarrheic and ST1 in non-diarrheic patients, but there was not seen a statistically correlation between subtypes and gastrointestinal (GI) symptoms like diarrhea. This finding is supported by previous studies showing unclear linkage between certain subtypes of *Blastocystis* and GI disorders (35, 36).

However, molecular study on distribution of *Blastocystis* subtypes among different human and animals populations is needed to clarify the rout of transmission and potential pathogenic role of *Blastocystis.*

## Conclusion

*Blastocystis* sp. is one of the most prevalent unicellular parasites among diarrheic and nondiarrheic patients and ST2 was the commonest subtype in positive samples particularly, those samples collected from diarrheic patients.
